# Nasal commensals reduce *Staphylococcus aureus* proliferation by restricting siderophore availability

**DOI:** 10.1093/ismejo/wrae123

**Published:** 2024-07-11

**Authors:** Yanfeng Zhao, Alina Bitzer, Jeffrey John Power, Darya Belikova, Benjamin Orlando Torres Salazar, Lea Antje Adolf, David Gerlach, Bernhard Krismer, Simon Heilbronner

**Affiliations:** Department of Infection Biology, Interfaculty Institute of Microbiology and Infection Medicine, University of Tübingen, 72076 Tübingen, Germany; Laboratory Medicine Center, The Second Affiliated Hospital, Nanjing Medical University, 210011 Nanjing, P. R. China; Department of Infection Biology, Interfaculty Institute of Microbiology and Infection Medicine, University of Tübingen, 72076 Tübingen, Germany; Cluster of Excellence EXC 2124 Controlling Microbes to Fight Infections, 72076 Tübingen, Germany; Department of Infection Biology, Interfaculty Institute of Microbiology and Infection Medicine, University of Tübingen, 72076 Tübingen, Germany; Cluster of Excellence EXC 2124 Controlling Microbes to Fight Infections, 72076 Tübingen, Germany; Department of Infection Biology, Interfaculty Institute of Microbiology and Infection Medicine, University of Tübingen, 72076 Tübingen, Germany; Cluster of Excellence EXC 2124 Controlling Microbes to Fight Infections, 72076 Tübingen, Germany; Interfaculty Institute of Microbiology and Infection Medicine, Institute for Medical Microbiology and Hygiene, UKT Tübingen, 72076 Tübingen, Germany; Department of Infection Biology, Interfaculty Institute of Microbiology and Infection Medicine, University of Tübingen, 72076 Tübingen, Germany; Cluster of Excellence EXC 2124 Controlling Microbes to Fight Infections, 72076 Tübingen, Germany; Department of Infection Biology, Interfaculty Institute of Microbiology and Infection Medicine, University of Tübingen, 72076 Tübingen, Germany; Cluster of Excellence EXC 2124 Controlling Microbes to Fight Infections, 72076 Tübingen, Germany; Interfaculty Institute of Microbiology and Infection Medicine, Institute for Medical Microbiology and Hygiene, UKT Tübingen, 72076 Tübingen, Germany; Ludwig-Maximilians-Universität München, Faculty of Biology, Microbiology, 82152 Martinsried, Germany; Department of Infection Biology, Interfaculty Institute of Microbiology and Infection Medicine, University of Tübingen, 72076 Tübingen, Germany; Cluster of Excellence EXC 2124 Controlling Microbes to Fight Infections, 72076 Tübingen, Germany; Cluster of Excellence EXC 2124 Controlling Microbes to Fight Infections, 72076 Tübingen, Germany; Interfaculty Institute of Microbiology and Infection Medicine, Institute for Medical Microbiology and Hygiene, UKT Tübingen, 72076 Tübingen, Germany; Ludwig-Maximilians-Universität München, Faculty of Biology, Microbiology, 82152 Martinsried, Germany; German Center for Infection Research “DZIF” partnersite Tübingen, Germany

**Keywords:** Staphylococcus aureus, nasal microbiome, iron limitation, siderophore, inter-species competition

## Abstract

The human microbiome is critically associated with human health and disease. One aspect of this is that antibiotic-resistant opportunistic bacterial pathogens, such as methicillin-resistant *Staphylococcus aureus*, can reside within the nasal microbiota, which increases the risk of infection. Epidemiological studies of the nasal microbiome have revealed positive and negative correlations between non-pathogenic species and *S. aureus*, but the underlying molecular mechanisms remain poorly understood. The nasal cavity is iron-limited, and bacteria are known to produce iron-scavenging siderophores to proliferate in such environments. Siderophores are public goods that can be consumed by all members of a bacterial community. Accordingly, siderophores are known to mediate bacterial competition and collaboration, but their role in the nasal microbiome is unknown**.** Here, we show that siderophore acquisition is crucial for *S. aureus* nasal colonization *in vivo*. We screened 94 nasal bacterial strains from seven genera for their capacity to produce siderophores as well as to consume the siderophores produced by *S. aureus.* We found that 80% of the strains engaged in siderophore-mediated interactions with *S. aureus.* Non-pathogenic corynebacterial species were found to be prominent consumers of *S. aureus* siderophores. In co-culture experiments, consumption of siderophores by competitors reduced *S. aureus* growth in an iron-dependent fashion. Our data show a wide network of siderophore-mediated interactions between the species of the human nasal microbiome and provide mechanistic evidence for inter-species competition and collaboration impacting pathogen proliferation. This opens avenues for designing nasal probiotics to displace *S. aureus* from the nasal cavity of humans.

## Graphical Abstract

**Graphical Abstract:**
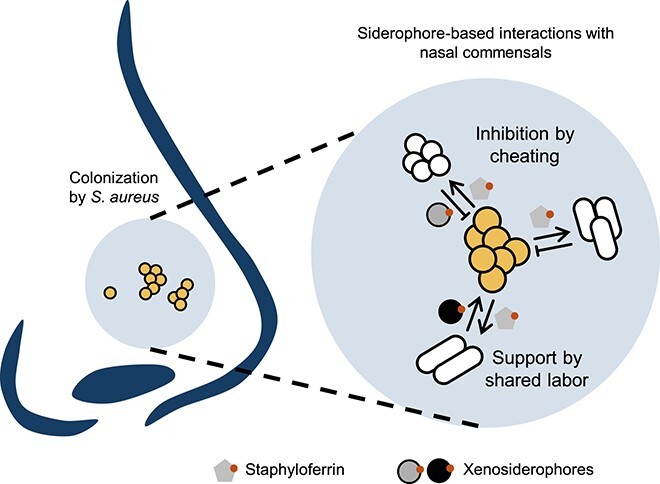

## Introduction

The human body is colonized by a multitude of bacteria from different genera that is collectively called the microbiome. The human microbiome is fundamentally associated with human health and disease. One aspect of this is that antibiotic-resistant bacterial pathogens can hide within the microbiomes of healthy individuals and can cause invasive disease if, e.g. they are transferred into surgical wounds after invasive interventions within hospitals [1]. *Staphylococcus aureus* is a prime example in this regard. The pathogen colonizes the anterior nares of approximately one-third of the human population, and colonization is a major risk factor for infection [2, 3]. *S. aureus* infections cause severe morbidity and mortality and can be difficult to treat as antibiotic-resistant lineages, such as methicillin resistant *S. aureus* (MRSA), are a worldwide concern. We know surprisingly little about the factors that determine whether an individual can be colonized by *S. aureus.* Host genetics and environmental conditions have only a moderate influence on *S. aureus* colonization [4, 5]. It is increasingly recognized that the presence of certain commensal species is important. Epidemiological analyses of nasal microbiomes showed the presence of *Finegoldia magna*, *Dolosigranulum pigrum*, and *Simonsiella* spp. to be negatively correlated with the presence of *S. aureus*, and corynebacteria are associated with a reduced absolute number of *S. aureus* cells [5, 6]. However, few studies have investigated the molecular interactions between *S. aureus* and other nasal commensals that underlie these observations. Production of antibacterial molecules is known to shape the composition of microbial communities [7] and has been shown to be important for the displacement of *S. aureus* by certain commensals [8–10]. In nutritionally limited environments, competitive or collaborative exploitation of natural resources is important in shaping microbial community structures [11, 12]. However, little is known about this in the context of the nasal microbiome [13]. A well-described mechanism of cooperation between bacterial cells is the secretion of “public goods” that are costly to produce but can be used by the entire community [14]. Iron-scavenging siderophores are a classical example. Siderophores are produced by many bacterial and fungal species and allow iron restriction in many environmental as well as host-associated habitats to be overcome. Due to their costly production, organisms called “cheats” that consume siderophores produced by others but lack endogenous production are well-known [15]. The presence of cheats reduces the fitness of producer cells and puts evolutionary pressure on siderophore biosynthesis/receptor genes to reduce such piracy [16]. Within the nasal cavity, host lactoferrin restricts the availability of free iron and iron-acquisition genes in *S. aureus* are induced [17]. Therefore, it seems plausible that siderophore production is of importance within this habitat. Additionally, the well-investigated routes of iron acquisition in *S. aureus* suggest adaption toward the presence of “xenosiderophores,” referring to siderophores produced by distantly related bacteria. *S. aureus* can produce and utilize two different siderophores, staphyloferrin A “SF-A” and staphyloferrin B “SF-B” [18, 19]. In addition, the bacterium is able to use hydroxamate and catecholate xenosiderophores, such as aerobactin, ferrichrome, or bacillibactin [20]. However, the extent to which *S. aureus* competes for siderophores with other members of the nasal microbiome is unknown. Similarly, it is unclear if siderophore piracy between *S. aureus* and certain commensals might explain positive or negative correlations between members of the human nasal microbiome.

We have investigated siderophore-based interactions between *S. aureus* and other members of the nasal microbiome. We assessed the ability of 94 nasal isolates from eleven different genera to produce siderophores and tested the ability of siderophore producers to support *S. aureus* growth. Additionally, we tested the ability of *S. aureus* to support the growth of siderophore-deficient species in a staphyloferrin-dependent manner. We found a plethora of siderophore-mediated interactions. Importantly, apart from *Corynebacterium propinquum*, most *Corynebacterium* spp. did not produce siderophores but were able to consume SF-A and/or SF-B. In co-cultivation experiments, we found that staphyloferrin piracy by competitors created a physiological burden for *S. aureus*, and their presence delayed its proliferation in an iron-dependent manner. Finally, we found iron chelating activity in the nasal cavity of human volunteers and demonstrated that siderophore acquisition is crucial for *S. aureus* to proliferate in the cotton rat model of nasal colonization, suggesting that siderophore-based competition or collaboration might be relevant for structuring the human nasal microbiome.

## Material and methods

### Chemicals

If not stated otherwise, reagents were purchased from Sigma.

### Bacterial strains, media, and culture conditions

A list of plasmid and bacterial strains used/generated is provided in Tables 1 and S1. Additionally, Table S1 provides information about the incubation time and media required for the different strains. In general, strains were streaked out on blood plates, either BM or TSA, and stored at 4°C. Antibiotics were added where appropriate: kanamycin (50 μg/ml), chloramphenicol (10 μg/ml), and streptomycin (250 μg/ml).

**Table 1:** Bacterial strains and plasmids.

**Strain/plasmid**	**Genotype/description**	**Source**
**Bacterial strains**		
*S. aureus* USA300 LAC	Wild type	[21]
*S. aureus* USA300 JE2	Plasmid-cured strain LAC	[22]
*S. lugdunensis* HKU09-01	Wild type	[23]
*S. lugdunensis* N920143	Wild type	[24]
*S. aureus* JE2 *fhuC::erm*	Strain NE406_SAUSA300_0633 of the Nebraska transposon mutant library	[22]
*S. aureus* Newman*^strepR^*	Streptomycin resistant wild type strain	[8]
*S. aureus* Newman^strepR^*fhuC::erm*	Contains the *fhuC::erm* mutation from NE406_SAUSA300_0633	This study
*S. aureus* Newman^strepR^*fhuC::erm* pRB473:*fhuC*	Plasmid-based complementation of *fhuC*	This study
*S. aureus* USA300 JE2 ∆*sbn*∆*sfa*	Markerless deletion of ∆*sfaDABC* and ∆*sbnABCDEFGHI*	This study
*S. aureus* USA300 JE2 *∆sfa*	Markerless deletion of ∆*sfaDABC*	This study
*S. aureus* USA300 JE2 *∆sbn*	Markerless deletion of ∆*sbnABCDEFGHI*	This study
*S. aureus* USA300 LAC::*sGFP*	sGFP fluorescence marker inserted between the genes *NWNM29-30*	[25]
*Corynebacterium hesseae* 10VPs_Sm8	Isolate from the nasal cavity of a human individual in Münster (Germany)	[26]
*C. hesseae* 10VPs_Sm8 Δ*R3O64_11615*	Markerless deletion of *R3O64_11615*	This study
*C. hesseae* 10VPs_Sm8 Δ*R3O64_03755*	Markerless deletion of *R3O64_03755*	This study
**Plasmids**		
pIMAY	Thermosensitive vector for allelic exchange	[27]
pRB473:*fhuC*	Expression of *fhuC* by its native promotor	This study
pJSC232	Kanamycin resistance/sucrose sensitivity vector for allelic exchange in *Corynebacteria* spp.	[28]

### Transduction of the *fhuC::erm* mutation

*Staphylococcus aureus* Newman^strpR^_*fhuC::erm* was created using phage transduction (phage Φ11) from the Nebraska transposon mutant library (strain NE406_SAUSA300_0633) into the *S. aureus* Newman^strpR^ background using standard protocols [29].

### Growth curve analysis of *S. aureus*

Staphylococcal strains were grown overnight in TSB at 37°C with agitation. Cells were harvested and washed with RPMI containing 1% casamino acids “CA” (Difco) and 10 μM ethylenediamine-di(o-hydroxyphenylacetic acid) “EDDHA” (fluorochem). OD_600_ was adjusted to 1, and 2.5 μl (strain Newman) or 5 µl (strains of USA300) were mixed with 500 μl of RPMI +1% CA + 10 μM EDDHA per well (final OD_600_ of 0.005 (Newman) or 0.01 (USA300)) in a 48-well microtiter plate (Nunc, Thermo Scientific). 200 ng/ml aerobactin (EMC Microcollections GmbH), 200 ng/ml ferrichrome (EMC Microcollections GmbH), 5.7% spent medium from USA300 JE2 Δ*sbn* containing SF-A, 9.1% spent medium from USA300 JE2 Δ*sfa* containing SF-B, 100 μg/ml holo-transferrin, or 20 μM FeSO_4_ were added as iron sources when appropriate. OD_600_ was measured every 15-30 min for 24 h in an Epoch2 reader (BioTek) or Tecan Spark microplate reader at 37°C orbital shaking.

### Construction of *sfaDABC* and *sbnH-I* deficient mutants

In-frame deletions in *S. aureus* were generated based on the technique described by Monk *et al.* [22]. The 500 bp sequences up- and downstream of the target genes were amplified using primers A/B and C/D, respectively (Table S2). The PCR fragments were fused by overlap extension PCR, cloned into pIMAY by restriction digestion, and used to transform *Escherichia coli* SA08B. Plasmids were confirmed by Sanger sequencing. The plasmids were then electroporated into USA300 JE2, and allelic replacement was performed using standard procedure [22]. Mutants were validated by PCR amplification and Sanger sequencing of the region of interest.

### Construction of *R3O64_11615* and *R3O64_03755* deficient *Corynebacterium hesseae* mutants

We constructed an in-frame deletion in *C. hesseae* as described previously [28]. Upstream and downstream sequences of the target genes were amplified with primers A/B and C/D, respectively (Table S2). The PCR products were inserted into the PstI-digested pJSC232 plasmid by sequence and ligation-independent cloning (SLIC) and then transferred into *E. coli* DH5α. The insertion was confirmed by DNA sequencing. The plasmids were subsequently electroporated into *C. hesseae*, and allelic replacement was performed using a kanamycin resistance marker as well as a negative selection marker for sucrose sensitivity [28]. Mutants were validated by PCR amplification and Sanger sequencing of the region of interest.

### Chrome azurol S overlay assay

The assay was performed as described earlier, with minor modifications [30]. For the assay, test organisms were grown for one week on BHI-agar plates containing 10 μM EDDHA and afterwards overlaid with CAS medium. CAS medium was prepared from four solutions, which were sterilized separately. Solution 1 (S1) was prepared by mixing 10 ml of 1 mM FeCl_3_*6 H_2_O in 10 mM HCl with 50 ml of an aqueous solution of chrome azurol S “CAS” (1.21 mg/ml) and 40 ml of an aqueous solution of hexadecyltrimetyl ammonium bromide “HDTMA” (1.82 mg/ml). Solution 2 (S2) contained Piperazine-1,4-bis(2-ethanesulfonic acid) “PIPES” at 30.24 g/L in 750 ml of a salt solution containing 0.3 g KH_2_PO_4_, 0.5 g NaCI, and 1.0 g NH_4_Cl. The pH was adjusted using 50% KOH to 6.8, then 15 g/L agar was added, and the volume was filled up to 800 ml. Solution 3 consists of 2 g glucose, 2 g mannitol, 493 mg MgSO_4_*7 H_2_O, 11 mg CaCI_2_, 1.17 mg MnSO_4_*H_2_O, 1.4 mg H_3_BO_3_, 0.04 mg CuSO_4_*5H_2_O, 1.2 mg ZnSO_4_*7H_2_O, and 1.0 mg Na_2_MoO_4_*2 H_2_O in 70 ml water. Solution 1–3 were autoclaved and then cooled to 50°C. 30 ml of Solution 4 (filter-sterilized) 10% (w/v) casamino acids were added and used to overlay the agar plates. After 4 h, the color of the plates was investigated. A change toward yellow/orange was considered as positive for siderophore production.

### Iron-depleted medium

To repress the siderophore-independent growth of microorganisms, iron-depleted RPMI was used. Therefore, two times concentrated RPMI media 1640 (LifeTechnologies, Burlington, ON, Canada) was reconstituted from powder in ddH_2_O, supplemented with 2% (w/v) CA, and treated with 7% (w/v) Chelex-100 resin (Bio-Rad, Mississauga, ON, Canada) at 4°C overnight. After sterile filtration, 20% of complement-inactivated horse serum (Sigma) and 20 μM EDDHA were added, and the solution was heated to 50°C. Separately, a 3% (w/v) agarose solution was prepared, autoclaved, and cooled to 50°C. Both solutions were mixed, and plates were poured (20 ml per plate). Plates containing horse serum are referred to as HS-RPMI.

### Reciprocal usage of siderophores

For the spot assay, nasal strains were cultured in TSB or BM liquid medium at 37°C for up to 7 days (see Table S1). Bacteria were harvested and washed two times with RPMI containing 10 μM EDDHA. Bacterial isolates suspected to consume siderophores were adjusted to OD_600_ = 0.05 and plated on HS-RPMI using a cotton swab. Siderophore producers were adjusted to OD_600_ = 4.0 and spotted on top (5 μl) of the dried background strain. The plates were incubated at 37°C for 24 h - 7 days (see Table S1).

### Plate-based co-cultures assay

Test strains were grown in TSB for 24 - 48 h (see Table S1). Bacteria were harvested and washed two times with RPMI containing 10 μM EDDHA. Strains were adjusted to 2 × 10^3^ CFU/ml, from which 100 μl were plated on RPMI-HS plates. Bacteria were added individually or mixed at a ratio of 1:1 and incubated for 24 h at 37°C. Bacterial colonies were imaged using a LEICA M125 microscope (1.25x magnification), and the diameter of the colonies was measured using Image J.

### Liquid-based co-culture assay

Staphylococcal strains were grown overnight (20 h) in TSB at 37°C with agitation. Cells were harvested and washed two times with RPMI containing 1% CA and 10 μM EDDHA. OD_600_ was adjusted to 1. For the co-cultivation, 5 μl of *S. auerus::sGFP* together with 5 μl of competing strain were mixed with 500 μl of RPMI+1% CA + 10 μM EDDHA+100 μg holo-transferrin per well (final OD_600_ of 0.02) in a 48-well microtiter plate (Nunc, Thermo Scientific). OD_600_ and fluorescence intensity (Ext._480 nm_/Em._525 nm_) were measured every 30 min for 24 h in a Tecan Spark microplate reader at 37°C orbital and linear shaking.

### Whole genome sequencing “WGS” and analysis

The genomes of *Corynebacterium simulans* 50MNs_SDm2, *Corynebacterium pseudodiphtheriticum* 90VAs_B3, *Corynebacterium hesseae* 10VPs_Sm8, *Bacillus cereus* 45MNs_B5, *Mammaliicoccus sciuri* 9VPs_Sm2, and *Citrobacter koseri* 44VAs_B2 were sequenced using long and short read sequencing.

For Illumina short-read sequencing, DNA was isolated from cell pellets using QIAGEN’s DNeasy PowerSoil Pro Kit according to the manual’s instructions with 2 min of vortexing in PowerBead Pro tubes. Libraries were prepared using the Illumina DNA Prep (M) Tagmentation kit according to the manufacturer’s protocol with 500 ng of DNA input and 5 cycles of indexing PCR. Libraries were checked for correct fragment length on an Agilent 2100 BioAnalyzer and pooled equimolarly. The pool was sequenced on an MiSeq v3 (Illumina), 600 cycles flow cell with a 2 × 150 bp read length and a depth of 70x genome coverage.

For long read sequencing, the cell pellet was resuspended in 600 μL of ATL buffer (Qiagen) and transferred to a ZR BashingBead Lysis Tube (Zymo Research). The tube was vortexed horizontally for 2 min on a vortex shaker. To optimize the DNA extraction, the supernatant was taken off and digested with RNAse A (Qiagen). The DNA was then automatically purified with the QIAamp 96 QIAcube HT kit (Qiagen) with additional proteinase K on a QIAcube HT following the manufacturer’s instructions. For library generation, the Ligation Sequencing Kit 109 (Oxford Nanopore) was used with native barcoding, following the manufacturer’s protocol, with 500 ng DNA input per sample and prolonged incubation times. Library size was assessed on a FEMTO Pulse (Agilent), and libraries were pooled equimolarly before sequencing on a FLO-PRO002 flow cell on a Nanopore PromethION device. 14.5 million reads with 55 GB were generated.

DNA sequence reads were assembled using nf-core pipeline bacass (v2.0.0 [31]). Assembled scaffolds were annotated using PGAP (NCBI, v.2021-11-29.build5742 [32]) and curated using the NCBI Genome Workbench (v3.7.0 [33]).

Homologous of *htsA* and *sirA* were identified using BLAST analysis. Biosynthetic gene clusters were predicted using antiSMASH 7.0 [34]**.**

### Phylogenetic comparison of nasal bacteria

The 16S rRNA locus was used to highlight the relatedness of the members of the nasal microbiome used in this study. The gene sequences for all species besides *C. hessae* were obtained from the NCBI Refseq or RiboGrove databases [35]. For *C. hesseae*, the respective locus was extracted from the WGS presented in this study. The resulting sequences were aligned using Clustal Omega, provided in the msa R package [36]. A phylogenetic tree was constructed using the UPGMA method provided by the phangorn package [37].

### Animal models and ethics statement

All animal experiments were conducted in strict accordance with the German regulations of the Gesellschaft für Versuchstierkunde/Society for Laboratory Animal Science “GV-SOLAS” and the European Health Law of the Federation of Laboratory Animal Science Associations “FELASA” in accordance with German laws after approval (protocol IMIT 1/15 for cotton rat colonization) by the local authorities (Regierungspräsidium Tübingen). All animal and human studies were carried out at the University Hospital Tübingen and conformed to institutional animal care and use policies. No randomization or blinding was necessary for the animal colonization models, and no samples were excluded. Animal studies were performed with cotton rats of both genders, 8–12 weeks old.

### Cotton rat nasal colonization

For the colonization, a spontaneous streptomycin-resistant mutant of *S. auerus* Newman wild type was selected on BM agar plates containing 250 μg ml^−1^ streptomycin. In this *S. aureus* Newman^strepR^, the cassette *fhuC::erm* was introduced using phage transduction from the Nebraska transposon mutant library (strain NE406_SAUSA300_0633). Cotton rats were anesthetized and instilled with either 1 × 10^7^ *S. aureus* Newman^strepR^ or 1 × 10^7^ *S. aureus* Newman^strepR^*fhuC::erm*. Five days after bacterial instillation, the animals were euthanized, and their noses were surgically removed. The noses were vortexed in 1 ml of 1 × PBS for 30 s. Samples were plated on agar plates containing 250 μg/ml streptomycin using an EddyJet 2W to determine the bacterial CFU. The plates were incubated for 2 days under aerobic conditions.

### Statistical analysis

Statistical analysis was performed by using GraphPad Prism (GraphPad Software, Inc., La Jolla, USA; version 9). Statistically significant differences were calculated by appropriate statistical methods as indicated. *P*-values of ≤.05 were considered significant.

### Measurement of iron chelating activity in the human nose

Both nostrils of healthy human volunteers were swabbed using a single sterile eSwab (Copan Diagnostics, Inc.). The swab was washed out in 300 μl PBS, and debris was removed by centrifugation. Afterwards, the commercially available SideroTec-HiSens assay kit (Accuplex Diagnostics) was used to assess the iron chelating activity using 100 μl of sample.

## Results

### Siderophore production among nasal bacterial species is diverse

It is known that *S. aureus* produces staphyloferrins but can also use siderophores produced by other species, “xenosiderophores”. We hypothesized that other nasal commensals might possess similar abilities, resulting in reciprocal dependencies.

**Figure 1:**
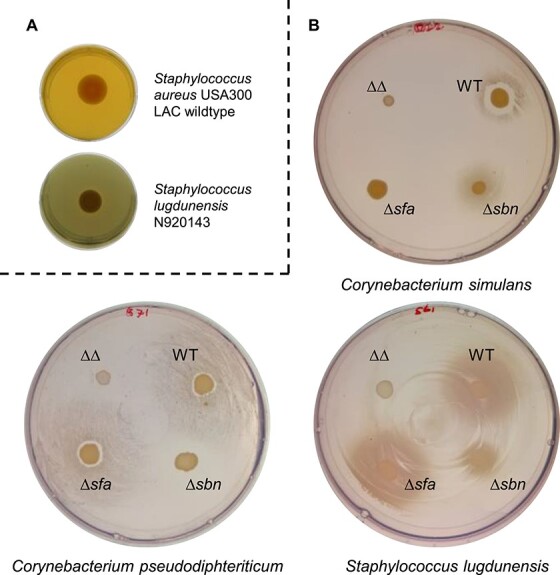
Siderophore production and staphyloferrin usage by nasal bacterial isolates. (A) Siderophore production: Bacterial isolates were spotted on BHI-EDDHA agar in 24 well plates and incubated for 1 week at 37°C**.** After incubation wells were overlaid with CAS-containing top agar. Color change indicates siderophore production and was assessed 4 h after the overlay. Shown are results for *S. aureus* USA300 LAC (positiv) and *S. lugdunensis* N920143 (negativ). (B) Staphyloferrin A and staphyloferrin B usage by nasal commensals: An even lawn of nasal commensal species was applied to iron-depleted RPMI plates with 10% horse serum. *S. aureus* USA300 strains producing either both staphyloferrins (WT), only staphyloferrin A (Δ*sbn*), only staphyloferrin B (Δ*sfa*), or none of the two (ΔΔ) were spotted on top of the lawn. Growth surrounding the strains was assessed after 24 h (*C. simulans* and *S. lugdunensis*) or 48 h (*C. pseudodiphtheriticum*) of incubation. Results of *C. simulans* (50Mns_SDm2), *C. pseudodiphtheriticum* (44VAs_Sa4), and *S. lugdunensis* (N920143), are shown.

**Figure 2:**
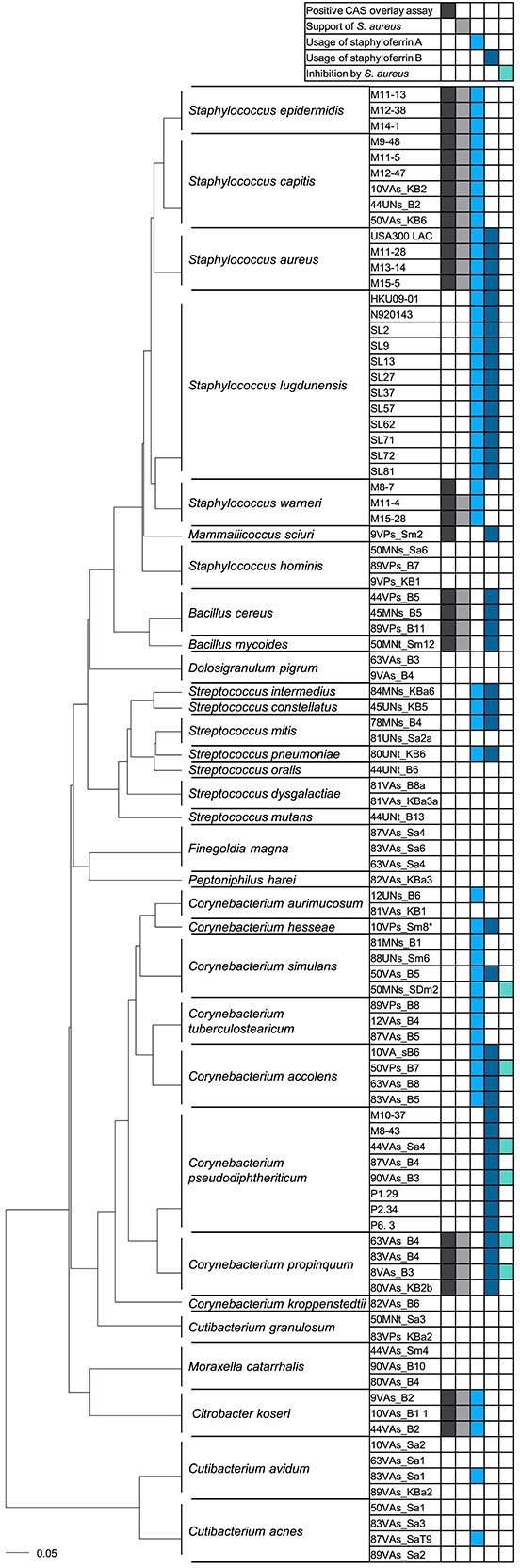
Summary of siderophore production and consumption. Bacterial isolates are grouped according to phylogentic similarity of the 16S rRNA gene. Siderophore production, is shown by dark gray. Growth improvement of *S. aureus* USA300 JE2 Δ*sbn*Δ*sfa* surrounding siderophore producers is indicated. Growth improvement of commensals by SF-A and SF-B as well as inhibition of commensals by *S. aureus* is shown. *The strain *C. hesseae* 10VPs_Sm8 was initially identified as a *C. aurimucosum* isolate.

To investigate this, we tested 94 bacterial isolates derived from the nasal cavity of human individuals in Münster (Germany) [26] as well as in Tübingen (Germany) [38]. The collection comprised species from 11 genera, namely *Staphylococcus*, *Bacillus*, *Citrobacter*, *Corynebacterium*, *Cutibacterium* (*Propionibacterium)*, *Dolosigranulum*, *Finegoldia*, *Mammaliicoccus, Moraxella*, and *Streptococcus*, including several strains from the same species. Siderophore production can be assessed using the chrome azurol S overlay assay “O-CAS” [30]. The assay indicates siderophore production by a color change to yellow (Figure 1A). *Staphylococcus aureus* USA300 LAC generates a yellow color due to the production of staphyloferrins, whereas *Staphylococcus lugdunensis* N920143 is unable to produce siderophores [39]. All strains in our collection were screened for siderophore production using this method (Fig. 2). Apart from *S. lugdunensis* and *Staphylococcus hominis*, all staphylococcal strains tested produced siderophores. This was expected as the locus *sfaDABC* encoding the genes for SF-A biosynthesis is highly conserved among staphylococci [18]. In addition, the *Bacillus*, *Citrobacter*, and *Mammaliicoccus sciuri* (formerly *Staphylococcus sciuri*) isolates also produced siderophores. Among the *Corynebacterium* isolates only *C. propinquum* produced siderophores. None of the other isolates, including *D. pigrum*, *F. magna*, *Moraxella catarrhalis*, *Peptoniphilus harei*, cutibacteria, and streptococci, showed positive reactions in the CAS assay.

### Nasal commensals profit from staphyloferrin-producing *S. aureus*

We speculated that nasal organisms lacking endogenous siderophore production might be able to acquire xenosiderophores. Staphylococci are frequent colonizers of the nasal cavity and prominent producers of siderophores. The majority of human-associated staphylococci, including *S. aureus* and coagulase-negative staphylococcal “CoNS” species, produce staphyloferrin A “SF-A”. In contrast, production of staphyloferrin B “SF-B” is a prominent trait of *S. aureus* but only rarely observed for CoNS [40–43]. To test if staphyloferrin-producing bacteria could promote the growth of nasal commensals, we constructed isogenic mutants of *S. aureus* USA300 JE2 lacking *sfaDABC* (no production of SF-A), *sbnA-I* (no production of SF-B), or a double mutant (no siderophore production). Only the double mutant was negative in the CAS assay, confirming complete loss of siderophore production (Fig. S1). Lawns of nasal commensals were applied to iron-limited agar plates containing 10% horse serum, “HS-RPMI.” The wild type *S. aureus* and the isogenic mutants were dotted on top of the lawn. After incubation, we often observed the growth of the commensals surrounding *S. aureus* WT or individual mutants being stimulated. In contrast, the *S. aureus* double mutant did not stimulate the growth of commensals (Figs 1B and 2). Enhanced growth around *S. aureus* colonies was frequently observed even when commensals (forming the lawn) produced siderophores endogenously. Most staphylococcal species profited from SF-A but not from SF-B production, as indicated by improved growth surrounding the *S. aureus ∆sbn* but not the *∆sfa* mutant (Fig. 2). *S. hominis* strains were the only staphylococcal species that did not benefit from SF-A or SF-B-producing *S. aureus*, whereas *S. lugdunensis* strains were the only staphylococci profiting from SF-A as well as from SF-B-producing *S. aureus* (Figs 1B and 2). This phenomenon was previously described for *S. lugdunensis* [39]. A prominent observation was that the production of SF-A and/or SF-B supported the growth of almost all *Corynebacterium* isolates. With the exception of a single *Corynebacterium aurimucosum* strain and *Corynebacterium kroppenstedtii*, all corynebacteria profited from either SF-A (7 of 27 strains), SF-B (12 of 27 strains), or both (6 of 27 strains). However, *Bacillus cereus*, *Bacillus mycoides*, *Citrobacter koseri*, *M. sciuri*, several streptococcal isolates, and individual strains of *Cutibacterium acnes* and *Cutibacterium avidum* were stimulated by staphyloferrin-producing *S. aureus* (Fig. 2). Altogether, these data indicate that staphyloferrins represent a widely accessible iron source for the bacterial species of the nasal cavity.

We also observed that the growth of individual corynebacterial strains was inhibited in close proximity to *S. aureus*, whereas growth was stimulated in a staphyloferrin-dependent manner with increasing distance (Figs 1B and 2). Analysis of the *S. aureus* USA300 JE2 genome revealed the presence of two putative bacteriocins with similarities to lactococcin and hyicin. These antimicrobial molecules might account for the observed effects.

### Staphyloferrin consumption by *Corynebacterium hesseae* is receptor dependent

**Figure 3:**
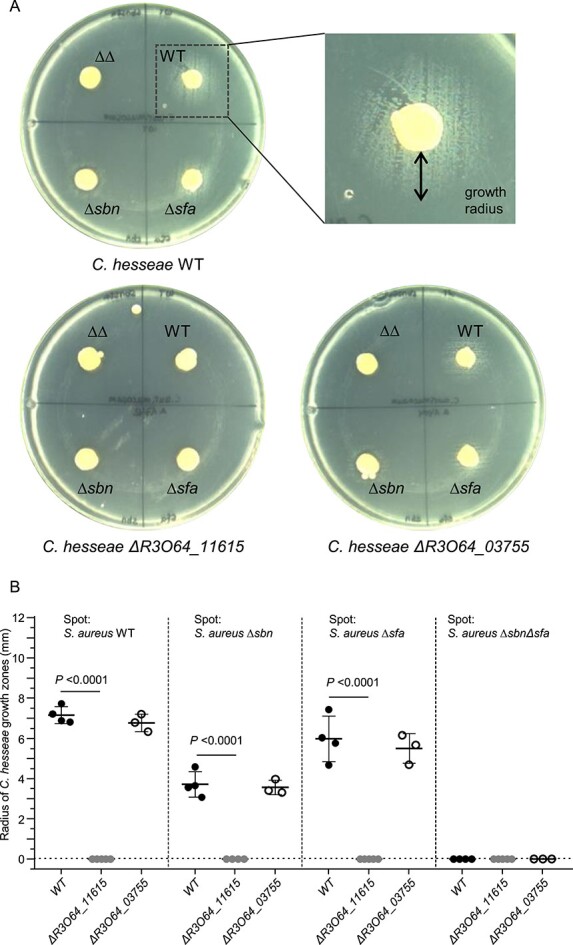
Staphyloferrin consumption by *Corynebacterium hesseae* 10VPs_Sm8. (A) Staphyloferrin consumption of *C. hesseae* is receptor dependent: An even lawn of *C. hesseae* (10VPs_Sm8 or its isogenic mutants Δ*R3O64_11615* and Δ*R3O64_03755*) were applied to iron-depleted RPMI plates. *S. aureus* USA300 producing either both staphyloferrins (WT), only staphyloferrin A (Δ*sbn*), only staphyloferrin B (Δ*sfa*), or none of the two (ΔΔ) were spotted on top of the lawn. Growth surrounding the spots (indicated by black arrow) was quantified 48 h post incubation. (B) Statistical analysis: Mean and SD of 3–4 independent experiments is shown. Statistical analysis was performed using ordinary one-way ANOVA (*P* < .0001) with subsequent multiple comparison.

*Staphylococcus aureus* uses the membrane-bound lipoproteins HtsA and SirA to acquire ferric forms of SF-A and SF-B, respectively [44–47]. We reasoned that nasal commensals might express homologous receptors enabling them to acquire staphyloferrins. To investigate this, we sequenced the genomes of six staphyloferrin-consuming strains from different species. We found genes encoding proteins with homology to HtsA and SirA in all strains (Table S3). However, the amino acid sequence identity of the staphylococcal receptors varied between 24% and 62%, which seemed too low to draw direct conclusions about the substrate specificity. To further investigate this, we focused on the SF-A and SF-B-consuming *Corynebacterium hesseae* strain 10VPs_Sm8, which we found amenable to genetic manipulation. The strain encodes the protein R3O64_11615 (43% and 26% identity to SirA and HtsA, respectively) as well as the protein R3O64_03755 (27% and 26% identity to SirA and HtsA, respectively). We created deletion mutants lacking these genes and tested their ability to thrive in the presence of *S. aureus* strains producing both, only one, or none of the staphyloferrins, respectively*.* We found loss of R3O64_11615 to be sufficient to abrogate growth promotion by SF-A as well as by SF-B-producing *S. aureus*, whereas loss of R3O64_03755 did not influence this phenotype (Fig. 3). This data suggests that SF-A and SF-B usage of *C. hesseae* is mediated by a single receptor. Additionally, these data demonstrate that the growth stimulation was solely caused by staphyloferrins and not by other metabolic products of *S. aureus*.

### *Staphylococcus aureus* profits from siderophore-producing commensals

**Figure 4:**
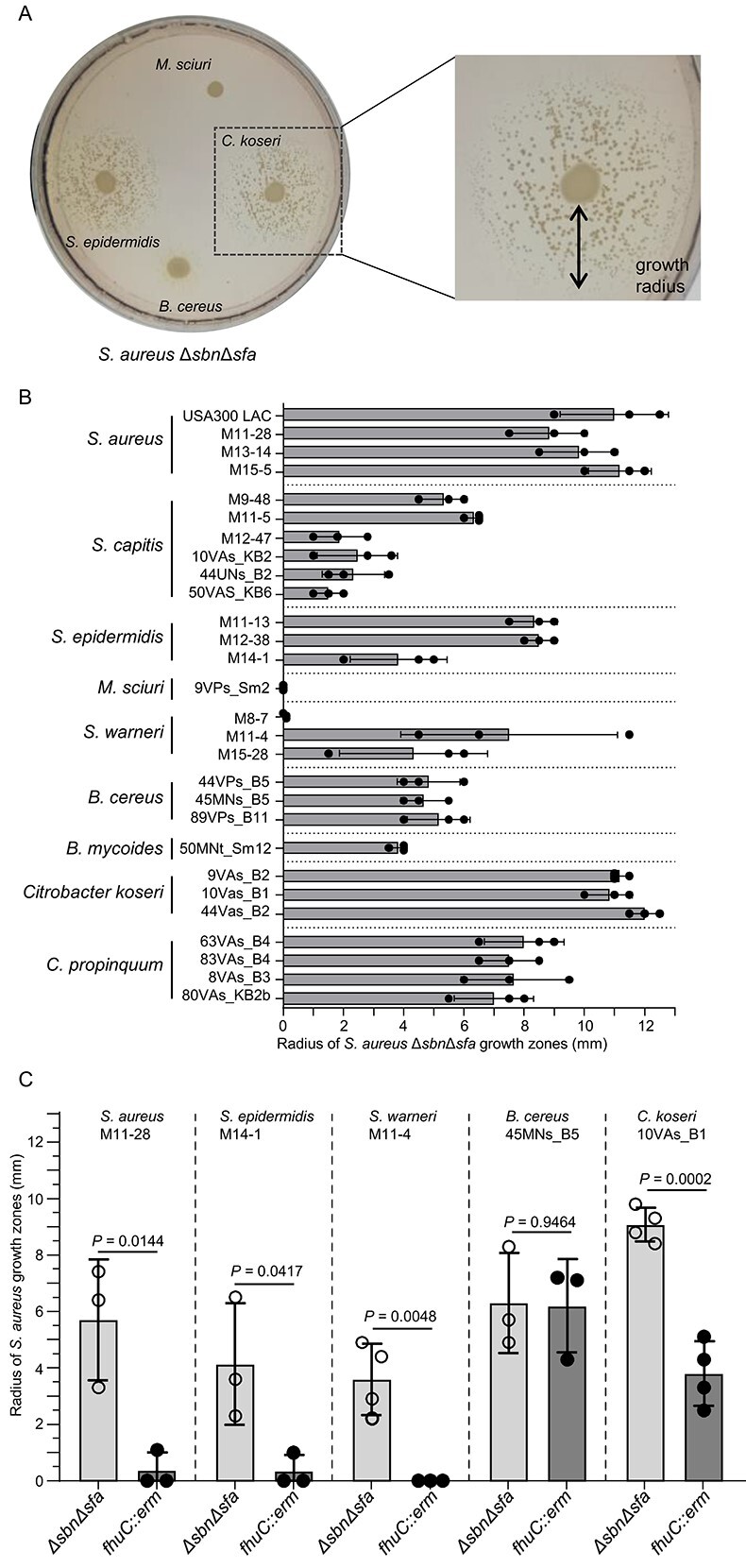
Usage of xenosiderophores by *S. aureus*. An even lawn of *S. aureus* USA300 JE2 Δ*sbn*Δ*sfa* or *fhuC::erm* was applied to iron-depleted RPMI plates with 10% horse serum. Siderophore-producing nasal isolates were spotted on top of the lawn. Growth surrounding the spots (indicated by black arrow) was quantified 17.5 h post incubation. (A) An example plate: Shown is *S. aureus* USA300 JE*2* Δ*sbn*Δ*sfa* with strains *S. epidermidis* M14-1, *C. koseri* 10VAs_B1, *B. cereus* 45MNs_B5, *Mammaliicoccus sciuri* 9VPs_Sm8. (B) Quantitative evaluation. *S. aureus* USA300 JE2 Δ*sbn*Δ*sfa* growth zones around different siderophore producers were measured. Mean and SD of 3 independent experiments is shown. (C) FhuC dependency: Growth zones of *S. aureus* USA300 JE2 Δ*sbn*Δ*sfa* and *fhuC::erm* surrounding the siderophore producers (*S. aureus* M11-28, *S. epidermidis* M14-1, *S. warneri* M11-4, *B. cereus* 45MNs_B5, and *C. koseri* 10VAs_B1) were assessed. Mean and SD of 3–4 independent experiments is shown. Statistical analysis was performed using an unpaired *t*-test. *P*-values are indicated.

*Staphylococcus aureus* can acquire xenosiderophores of the hydroxamate-type via the FhuABCD1/D2 system and catecholate type siderophores via SstABC. Therefore, we speculated that *S. aureus* might profit from the presence of the identified siderophore-producing strains. To test this, we plated even lawns of *S. aureus* ∆*sfa*∆*sbn* on iron-limited HS-RPMI plates, spotted siderophore-producing nasal isolates on top of the lawn, and assessed *S. aureus* growth surrounding the inocula (Figs 4A and 2). The ∆*sfa*∆*sbn* mutant does not secrete staphyloferrins, but all siderophore receptors are expressed. All *S. aureus* isolates fostered the growth of the ∆*sfa*∆*sbn* mutant, which is consistent with the production of SF-A and SF-B. *C. propinquum* and *C. koseri* isolates promoted proliferation of the ∆*sfa*∆*sbn* mutant, suggesting that these strains produce siderophores that are accessible to *S. aureus*. Most CoNS isolates also allowed the growth of *S. aureus* ∆*sfa*∆*sbn*, which is consistent with the previous finding that staphylococci carry SF-A biosynthesis genes [18]. Finally, the *B. cereus* and *B. mycoides* isolates allowed proliferation of *S. aureus* ∆*sfa*∆*sbn*. In contrast, individual isolates of *Staphylococcus warneri and M. sciuri* did not allow proliferation of the *S. aureus* mutant, suggesting that their siderophores are not accessible to *S. aureus*. We analyzed the genome sequences of selected producers to identify their siderophores (Table S4). *C. koseri* 44VAs_B2 encoded the biosynthesis genes for aerobactin (mixed type), turnerbactin (catecholate type), and yersiniabactin (phenolate type). Aerobactin has been shown before to support *S. aureus* growth via the FhuABCD1/D2 system [20, 48], and turnerbactin might be acquired via the SstABC system [48]. *B. cereus* 45MNs_B5 encoded genes for the synthesis of the catecholate-type siderophores bacillibactin and petrobactin, which might be acquired by *S. aureus* via the SstABC system. In contrast, *M. sciuri* 45MNs_B5, which did not support *S. aureus* proliferation, encoded a single, yet uncharacterized, siderophore biosynthesis cluster.

FhuC serves as a housekeeping ATPase that energizes the acquisition of SF-A, SF-B, and hydroxamate siderophores [18, 20, 49]. The catecholate acquisition system SstABC of *S. aureus* is independent of FhuC [48]. We used a *fhuC::erm* mutant of *S. aureus* USA300 JE2 [22] to verify that the enhanced proliferation of *S. aureus* surrounding the commensal was caused by the provision of xenosiderophores. Enhanced growth of the *fhuC*-deficient mutant surrounding *S. aureus*, *S. epidermidis*, and *S. warneri* strains was not observed (Fig. 4C), further strengthening the idea that staphyloferrins were responsible for growth stimulation. Proliferation of the *fhuC*-deficient strain surrounding *C. koseri* was markedly reduced, whereas growth surrounding *B. cereus* was independent of FhuC (Fig. 4C). This data agrees with *C. koseri* producing both hydroxamate and catecholate type siderophores, whereas *B. cereus* produces exclusively catecholate-type siderophores, which are acquired independently of FhuC. These experiments confirm that the growth enhancement is dependent on the receptor-specific exchange of siderophores between *S. aureus* and nasal commensals. Additionally, the data show that increased proliferation of *S. aureus* surrounding nasal commensals depends on its ability to import the produced siderophores.

### Classification of siderophore-based interactions between *S. aureus* and nasal commensals

**Figure 5:**
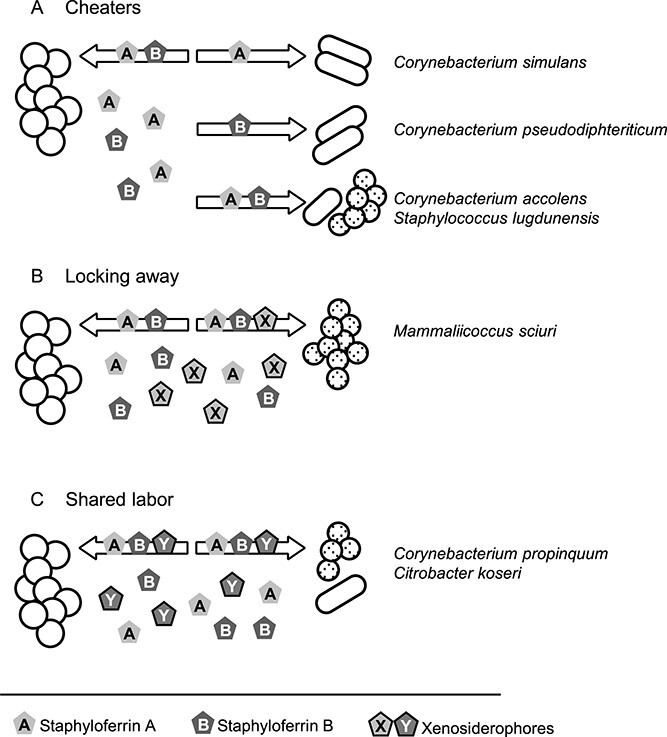
Schematic diagram of siderophore-based interactions between *S. aureus* and nasal commensals. (A) “Cheater” phenotype. Commensals without endogenous siderophore production consume staphyloferrins. (B) “Locking away” phenotype. Commensals consume staphyloferrins, whereas endogenous siderophores of the commensals are NOT accessible to *S. aureus*. (C) “Shared labor” phenotype. Siderophores produced by *S. aureus* and the commensals are reciprocally accessible.

Our experiments show that *S. aureus* interacts with nasal commensals in three distinct fashions (Fig. 5). First, several strains did not produce siderophores but consumed staphyloferrins as “cheaters.” Most corynebacterial isolates belong to this category (Fig. 5A). Second, certain commensal species produce siderophores that are inaccessible to *S. aureus* while simultaneously consuming staphyloferrins are consumed. This interaction is referred to as “locking away” [16] (Fig. 5B). *M. sciuri* isolate is a prominent example of this type. Third, several commensals produced siderophores that supported *S. aureus* growth, while staphyloferrins were consumed by the commensal “shared labor” (Fig. 5C). *C. koseri* and *C. propinquum* belong to this category.

Siderophore production is metabolically costly, and it is known that siderophore-based interactions shape bacterial communities [15, 16]. In particular, competitors with the “cheater” and “locking away” phenotypes decrease the relative fitness of siderophore producers in bacterial communities. This suggests that certain nasal commensals might decrease the fitness of *S. aureus* in iron-limited environments.

### Staphyloferrin consumption by competitors reduces fitness of *S. aureus*

**Figure 6:**
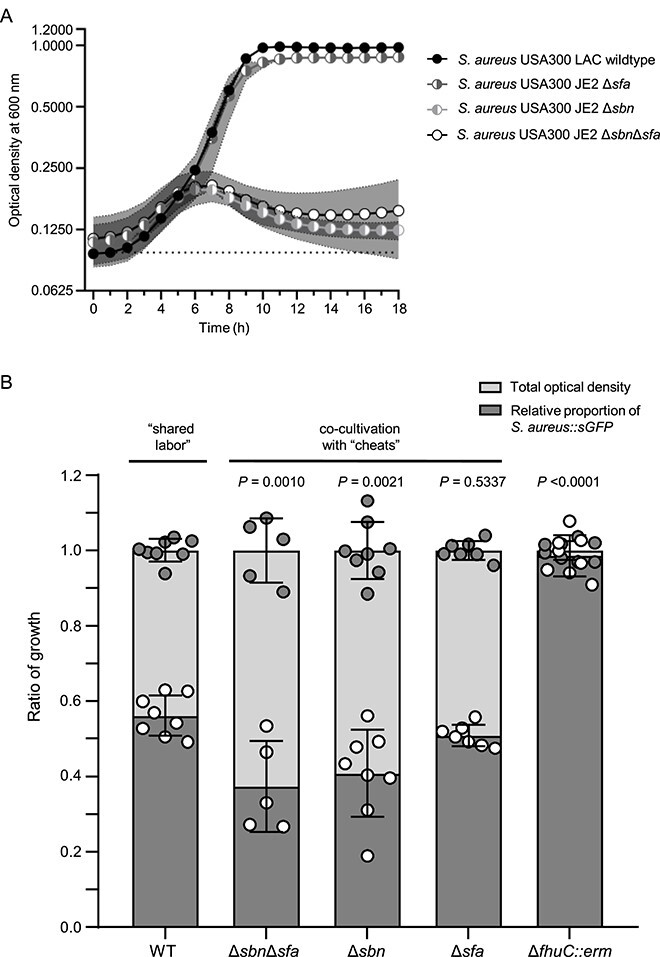
Growth and co-cultivation in iron-limited medium. (A) Growth curves in iron-limited medium. *S. aureus* USA300 LAC wildtype and *S. aureus* USA300 JE2 strains lacking either the staphyloferrin A and B or both production genes were inoculated to an optical density of 0.01 and grown for 18 h in 500 μl iron-limited medium (1x RPMI, 1% casamino acid, 10 μM EDDHA, 100 μg holo-transferrin) at 37°C under constant shaking in the Tecan spark 10M multimode microplate reader. Growth was monitored via optical density at 600 nm. Shown is the mean of 3–10 biological replicates of each strain. The dotted line indicates the media control. (B) Co-cultivation in iron-limited medium. For each co-cultivation the strain *S. aureus::sGFP* was mixed in equal portions (start OD of 0.02) with *S. aureus* USA300 LAC wildtype, *S. aureus* USA300 JE2 lacking staphyloferrin production genes (Δ*sbn and/or* Δ*sfa*) or *S. aureus* USA300 JE2 defective in its siderophore uptake systems (*fhuC::erm*). The strains were grown for 24 h in 500 μl iron-limited medium at 37°C under constant shaking in the Tecan Spark 10M multimode microplate reader. Growth was monitored via optical density at 600 nm and fluorescence intensity of the GFP signal (Ext. 480 nm and Em. 525 nm). The fluorescence values were calculated from the optical density values by using the equation y = 12.54 + 1284*x (*R*^2^ = 0.98). The calculated values are shown in dependence of the measured optical density values at timepoint of 10 h, which were set to 1. Statistical analysis was performed using ordinary one-way ANOVA (*P* < .0001) with subsequent multiple comparison by comparing all relative proportions of *S. aureus::sGFP* of each mixed culture with the shared labor condition.

**Figure 7:**
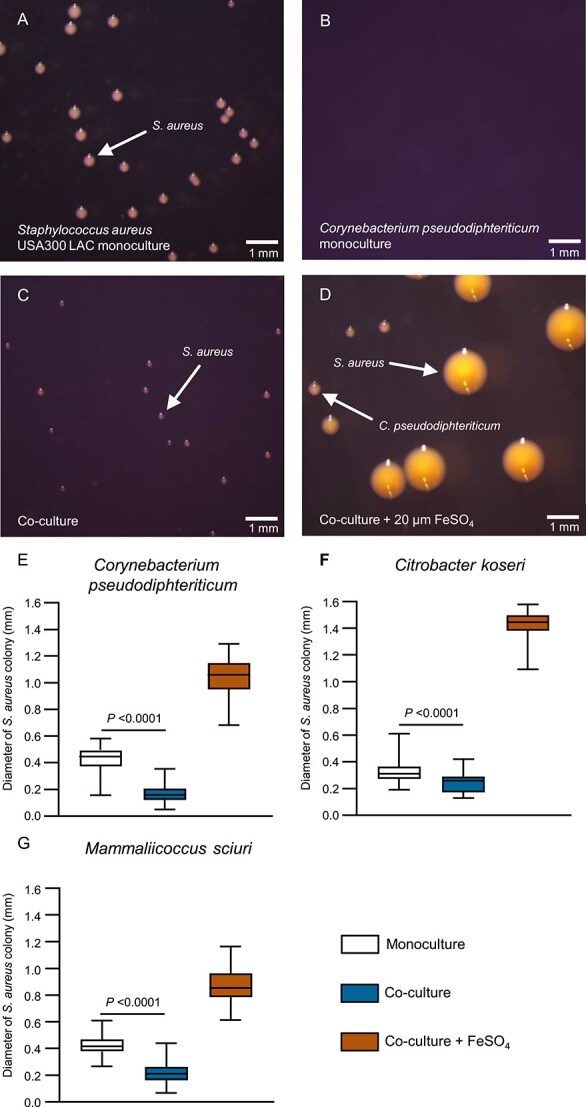
Siderophore-based interactions with nasal commensals impact growth of *S. aureus*. (A–E) Impact of commensals on *S. aureus* colony sizes. *S. aureus* USA 300 LAC wildtype and *C. pseudodiphtheriticum* strain 90VAs_B3 were either plated individually (A+B), or mixed in even numbers (C-D) and plated on HS-RPMI plates (A-C) or HS-RPMI plates supplemented with 20 μM FeSO_4_ (D) after 24 h incubation at 37°C, pictures of the plates were taken and the diameter of *S. aureus* colonies was measured using ImageJ. Representative pictures are shown. (E–G) Summary of *S. aureus* USA300 LAC colony sizes under the various conditions. Effects of coculture with *C. pseudodiphtheriticum* strain 90VAs_B3, *C. koseri* 44VAs_B2 and *Mammaliicoccus sciuri* 9VPs_Sm2 are shown*.* Data represent the diameter of 44-232 individual colonies from three independent experiments. Floating bars represent 25th and 75th percentiles, the horizontal lines represent the medians. Whiskers show minimum and maximum range. Statistical analysis was performed using unpaired Mann–Whitney *t*-test. *P*-values are indicated.

We designed a physiological assay to investigate if siderophore consumption by competitors might impact the proliferation of *S. aureus* USA300 LAC. The staphyloferrin-producing wild type strain was able to proliferate in iron-deficient RPMI with holo-transferrin as a sole source of iron, whereas the Δ*sfa*Δ*sbn* mutant did not grow (Fig. 6A). Further, the Δ*sfa* mutant showed normal levels of growth, but the mutation of *sbn* abrogated proliferation. This indicates that staphyloferrin B is crucial under these experimental conditions (Fig. 6A). This is explained by the fact that the exponential growth of *S. aureus* in the presence of glucose is based on fermentation [50, 51]. Previous studies showed that SF-B production is dominant over SF-A production during fermentation due to a dedicated citrate synthase that allows SF-B production even when the activity of the TCA cycle is low [52, 53]. For competition experiments, we used a *S. aureus* USA300 LAC expressing sGFP [25]. This did not influence the growth rate of the strain (Fig. S2A) but produced a clear fluorescent signal in linear proportion to the optical density of the culture (Fig. S2B and C). We mixed the WT *S. aureus* expressing sGFP with unlabeled *S. aureus* WT or staphyloferrin-deficient mutants in an equal ratio to reflect conditions of “shared labor” and “staphyloferrin cheating,” respectively. An analysis of the GFP/OD correlation was used to assess the proportion of both strains at the end of the experiment. “Shared labor” resulted in even ratios of GFP positive to GFP negative cells (Fig. 6B), indicating that both strains grew equally well. In contrast, competition with the Δ*sbn*Δ*sfa* and Δ*sbn* mutants allowed similar OD values to be reached but reduced the GFP signal of the mixed culture, suggesting decreased fitness of the WT compared to the cheats. Competition with the SF-A-deficient strain did not affect the fitness of the WT, which correlates with the observation that SF-A production was dispensable under these conditions. To further confirm the relevance of siderophore sharing, we included the *fhuC*-deficient strain (*S. aureus* USA300 JE2 *fhuC::erm*). The strain is able to produce SF-A and SF-B but fails to acquire them. The *fhuC::erm* mutant was completely displaced by the GFP-positive WT strain by the end of the experiment, highlighting the relevance of siderophore acquisition in the competition experiment. Together, these experiments show that siderophore usurpation by competitors reduces the competitive fitness of staphyloferrin-producing *S. aureus* strains*.* In this light, we hypothesized that the presence of nasal isolates that consume staphyloferrins or provide xenosiderophores might impact the growth of *S. aureus* during co-cultivation. Nasal commensals failed to grow under the liquid co-culture conditions described above. Therefore, we used agar plate-based experimental conditions and chose individual nasal strains showing different interactions with *S. aureus*. First, we chose the “cheater” *C. pseudodiphtheriticum*, which consumes SF-B, and investigated its effects on *S. aureus* USA300 JE2. Mono as well as mixed cultures (1:1 ratio) were plated on iron-limited HS-RPMI, and the size of *S. aureus* colonies was measured after 24 h of incubation (Fig. 7). *S. aureus* formed regular sized colonies in monoculture (mean colony size of 0.4188 mm), but *C. pseudodiphtheriticum* was unable to form colonies under these conditions. During co-culture, the size of the *S. aureus* colonies was reduced by 60% (mean 0.168 mm), although *C. pseudodiphtheriticum* colonies were hardly detectable. To verify that this effect was caused by competition for iron, we included 20 μM FeSO_4_ into the plates, which increased the mean colony size of *S. aureus* in co-culture to 1.045 mm. This represents an increase of 160% above the level achieved in monoculture in the absence of additional iron. The additional iron also allowed *C. pseudodiphtheriticum* to form visible colonies. This strongly suggests that iron limitation is the dominant driver of *S. aureus* colony size in this assay. We speculated that species “sharing labor” with *S. aureus* by providing accessible siderophores might not impact *S. aureus* colony formation. To test this, we used *C. koseri* (strong support of *S. aureus*, Fig. 4) and repeated the co-culture experiment. We found that the presence of. *C. koseri* reduced *S. aureus* colony size only slightly (16% reduction), suggesting less fierce competition between the species. *M. sciuri* produces a siderophore that is not accessible to *S. aureus* while simultaneously consuming SF-B (locking away interaction). Co-culture reduced the size of *S. aureus* colonies by 49%. This is an indication of strong competition, but the effect was not more severe than that observed for co-culture with *C. pseudodiphtheriticum.*

### Siderophore acquisition is needed for nasal colonization by *S. aureus*

**Figure 8:**
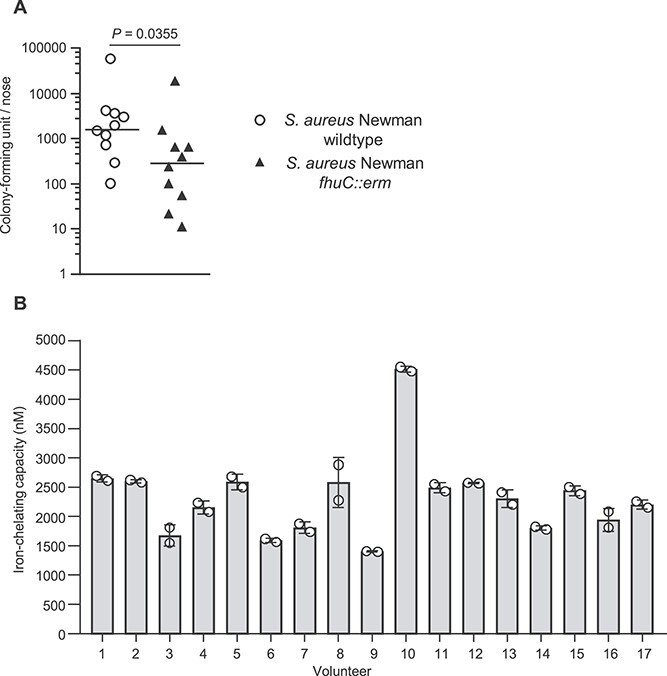
Siderophore acquisition is important for nasal colonization of cotton rats and iron-chelating activity is identified in human nasal specimen. (A) Cotton rat nasal colonization: 1 × 10^7^ CFU of *S. aureus* strain Newman^strepR^ and the isogenic *fhuC::erm* mutant were inoculated into the nares of 8–12 week old cotton rats. Five days post inoculation the animals were sacrificed, noses were harvested and *S. aureus* CFU were enumerated. Statistical analysis was performed using unpaired Mann–Whitney *t*-test. *P*-value is indicated. (B) Siderophore level in the human nose: Nasal specimens of 17 random volunteers were collected and the iron-chelating activity was determined using the SideroTec-HiSens Assay (Accuplex Diagnostics Ltd.). Shown are the mean and SD of two technical replicates for each volunteer.

To investigate the relevance of siderophore acquisition to the ability of *S. aureus* to colonize the nares of cotton rats, we replaced the wild-type *fhuC* gene of a streptomycin resistant-mutant of *S. aureus* Newman with a *fhuC::erm* mutation [22]. As described previously, the *fhuC::erm* mutant grew normally in the presence of FeSO_4_ but was strongly disabled in iron-limited medium even if aerobactin (a substrate of FhuBGD_1_) was added. Similarly, the mutant grew poorly when culture supernatants of *S. aureus* Δ*sfa* or Δ*sbn* were added as sources of SF-B or SF-A, respectively, which has been described before [54]. All phenotypes could be complemented by plasmid-based expression of *fhuC* (Fig. S3). We used the cotton rat model to investigate the relevance of siderophore acquisition for nasal colonization. Compared to the WT, the level of colonization was significantly reduced when the *fhuC::erm* mutant was implanted (Fig. 8A). This confirmed the iron-limited environment within the nasal cavity and shows that siderophore acquisition is relevant for *S. aureus* to overcome the nutritional iron limitation within the nasal cavity.

### Siderophores are produced in the nasal cavity of humans

We sampled the nasal cavities of 17 human volunteers and used the Sidero-Tec-HiSens Assay to assess the iron-chelating activity. The measured iron-chelating capacity suggested siderophore concentrations of 1.2–4.4 μM (Fig. 8B), highlighting that production and most likely competition for siderophores are relevant within the human nasal microbiome.

## Discussion

The production of iron-binding siderophores facilitates the acquisition of nutritional iron and is of enormous biological importance for bacteria. Siderophores are produced by bacteria that have been isolated from many different environmental habitats, including sea and fresh water [55, 56], soil [30, 57], and also from bacteria living in association with multicellular organism, including plants and animals [58, 59]. Siderophores are important facilitators of microbial interactions. They are secondary metabolites that are resource-intensive to produce. Upon secretion, siderophores represent public goods that are accessible to the entire microbial community, and many bacterial species possess receptors for such xenosiderophores [20, 39, 60, 61]. Several mechanisms of positive and negative interference have been described [16]. Acquisition of xenosiderophores is usually of benefit to the consumer, as iron needs are satisfied without the costly production of siderophores [39]. However, sole dependence on xenosiderophores harbors the risk of high dependency on other bacteria. In fact, it has been demonstrated that the growth of otherwise “unculturable bacteria” can be promoted by siderophores of co-occurring species, exemplifying the existence of organisms that are fully dependent on siderophores produced by others [62]. Regarding the producer organism, consumption of siderophores by co-occurring non-producers reduces fitness, as the benefits of the costly production are shared with others [15, 63]. In contrast, some bacteria can reciprocally exchange siderophores “shared labor,” which will not skew competitive fitness. Finally, the production of uncommon siderophores can be advantageous for the producer, because no putative competitor can benefit from its production. In this case, the siderophore will cause increased iron restriction for the non-producer “locking away” [64, 65]. Accordingly, siderophore-based interactions can foster competition as well as collaboration and influence the structure of bacterial communities. These ecological concepts are frequently studied in environmental communities [15]. However, it is becoming more evident that they also apply to microbiomes and can be associated with the susceptibility of the host towards pathogen colonization and infection, a concept that applies to plant as well as animal pathogens [59, 61, 66]. In humans, the immune system potentiates natural iron restriction by producing iron-chelating molecules, a phenomenon called nutritional immunity. Accordingly, siderophores are virulence factors enabling the proliferation of pathogens in normally sterile tissue [67]. However, nutritional immunity is also acting on mucosal and skin surfaces. Iron-binding lactoferrin is found in human secretions including sweat, tears, and breastmilk, as well as gastric, pancreatic, and nasal secretions [68], strongly suggesting that microbial communities colonizing human body surfaces face severe iron limitation. In fact, characterization of the human gut microbiome showed that changes in nutritional iron intake impose drastic effects on its composition, strongly suggesting that iron availability determines the success of species in polymicrobial communities [58]. This is supported by the finding that the probiotic *E. coli* Nissle strain displaces the pathogenic *Salmonella enterica* serovar Typhimurium in a siderophore-dependent manner [66]. Similarly, xenosiderophore usage is crucial for *Bacteroides thetaiotamicron* to grow in the context of the inflamed gut [61]. Little is known regarding the importance of iron for the human nasal microbiome. However, gene expression analysis of *S. aureus* isolated directly from human as well as from cotton rat nasal cavities has shown that iron acquisition genes are expressed during colonization, suggesting that the bacteria experience iron restriction [17, 69, 70]. In line with this, we showed here that iron-chelating activity is detected within the nasal cavity of humans and that a *fhuC*-deficient *S. aureus* mutant was attenuated during nasal colonization of cotton rats. This shows the importance of siderophore production and acquisition in the nasal cavity. A *fhuC-*deficient strain is still able to acquire catecholate-type xenosiderophores, highlighting that a reduction in the acquirable spectrum of siderophores reduces the fitness of *S. aureus*. A similar siderophore-dependence during nasal colonization of mice has been observed for *Klebsiella pneumonia* [71], suggesting a broad relevance of nutritional immunity for reducing pathogen colonization. The network of siderophore-based interactions within the nasal microbiome is unclear. We found that about one-third of nasal isolates produce siderophores, with the dominant producers being staphylococci along with *C. propinquum*, *C. koseri*, and *B. cereus*. However, *Citrobacter* and *Bacillus* are not regarded as frequent nasal commensals. Most likely, these species are intermittently introduced to and lost from the nasal microbiome, and their role in shaping communities by providing siderophores is therefore unclear. A striking finding of our experiments is that corynebacterial species were prominent consumers of SF-A or SF-B or both. According to the ecological considerations detailed above, this “cheating” phenotype should be associated with negative effects for staphyloferrin producers, which we confirmed *in vitro*, and it can be assumed that these interactions are relevant *in vivo*. Studies investigating the composition of the human nasal microbiome have frequently reported that the presence of *Corynebacterium* spp. is associated with decreased absolute numbers of colonizing *S. aureus* [5, 72, 73]. This might, in part, be due to siderophore cheating. A correlation between *S. aureus* and *C. pseudodiphtheriticum* was reported previously [6], and we found *C. pseudodiphtheriticum* to profit from SF-B, which might explain its frequent association with *S. aureus* in human nasal communities. Human trials showed that instillation of corynebacterial species (among them *C. pseudodiphtheriticum)* into the nostrils of human volunteers reduced or even abolished *S. aureus* colonization [74, 75], suggesting that the species are able to interfere with *S. aureus* colonization *in vivo*. However, to determine if interference is caused by siderophore cheating awaits experimental validation. In our experiments, the only corynebacterial species producing a siderophore was *C. propinquum*. This siderophore was previously described as dehydroxynocardamine and was found to inhibit the growth of *S. epidermidis*, most likely by reducing the availability of nutritional iron*.* In contrast, *S. aureus* was not affected by dehydroxynocardamine [65]. We found that *S. aureus* profited from *C. propinquum*, whereas *C. propinquum* profited from SF-B but not from SF-A production. This suggests a reciprocal adaption between *S. aureus* and *C. propinquum*, resulting in “shared labor” phenotypes. In contrast, *S. epidermidis* and *C. propinquum* engage in a “locking away” type of interaction as the siderophores are reciprocally inaccessible. In the experiments reported here, we identified multiple strains of different species and genera that interacted with *S. aureus* in diverse fashions (shared labor/cheat/locking away). We found that co-culture under iron-limited conditions always resulted in *S. aureus* having a decreased colony size. Most likely this reflects the competition for essential nutrients, including carbon and energy sources, which are all limited by the experimental conditions employed. However, the “cheating” and “locking” away phenotypes had a more pronounced impact on *S. aureus* colony sizes than “shared labor,” which is in line with general ecological principles [16]. In a co-submitted manuscript by Rosenstein *et al.*, we demonstrate siderophore-based interactions between *S. epidermidis* and *S. lugdunensis* that can, at least partly, explain the positive correlation of the species within the nasal microbiome of humans (available as a preprint at https://doi.org/10.1101/2024.02.29.582731). In many instances, siderophore consumption and production phenotypes were strain-rather than species dependent. Similarly, it has been reported that corynebacteria inhibit *S. aureus* strains with different *agr* alleles to variable degrees [72]. This phenotypic heterogeneity suggests that any antagonizing/stimulating effects might be dependent on the genotypes of particular strains, and extrapolation of findings to the species level might not always be valid. This has also to be considered for other traits that influence bacterial interactions, such as bacteriocin-production, which is frequently strain-dependent [7]. Similarly, we found that individual corynebacterial isolates were inhibited in close proximity to *S. aureus*. It has been shown that the targeted killing of *S. aureus* by *P. aeruginosa* releases nutritional iron to support the growth of *P. aeruginosa* [76], and it seems plausible that *S. aureus* specifically targets siderophore cheaters within the nasal microbiome to reduce competition for iron. We showed recently that the staphylococcal bacteriocin Epilancin, which is produced by many different staphylococcal species, is specifically targeting corynebacterial strains [77]. However, the additional layer of complexity added by bacteriocin-dependent killing on siderophore-based interactions is beyond the scope of this manuscript and needs further experiments. The experiments performed herein show a high diversity of siderophore-based interactions. Staphyloferrins were shown to be important siderophores that are consumed by strains of different species and genera. This suggests that commensals can create a hostile environment for staphylococci by siderophore cheating. However, it remains unclear to what extent siderophore-based interactions influence the composition of the nasal community *in vivo*. We did not perform co-colonization experiments using cotton rats as this model relies on animals harboring a rodent nasal microbiome that is not comparable to humans [78]. Animals raised in our facility carry staphylococci and corynebacterial species along with *E. coli*, *Enterobacter cloacae*, *Enterococcus faecalis*, *Rothia nasimurium*, and *Bacillus megaterium* (Table S5). Several of these strains produce siderophores (Table S5) that would distort the experiments and prevent meaningful interpretation. In future studies, to investigate the interactions within the nasal microbiome, the development of a colonization model in gnotobiotic (germ-free) animals is needed to allow the creation of humanized nasal microbiomes *in vivo.*

We have demonstrated a multitude of diverse siderophore-dependent interactions among members of the nasal microbiome. Several corynebacterial species consume staphyloferrin B, which is predominantly produced by *S. aureus* and rarely by CoNS, showing a particular adaption of the former to the presence of the potential pathogen. Siderophore piracy reduces the fitness of the producer. Accordingly, our data provide a mechanistic explanation of the frequently observed reduced level of *S. aureus* colonization in individuals carrying high numbers of corynebacteria and pave the way for the development of nasal probiotics to reduce *S. aureus* colonization in humans.

## Supplementary Material

Siderophores_Supplementary_Figures_Corrected

## Data Availability

Sequence data that support the findings of this study have been deposited as BioProject to NCBI with the primary accession PRJNA1028639. Other data are provided within the manuscript or supplementary information files.
