# Supplementary material for: Nasal commensals reduce Staphylococcus aureus proliferation by restricting siderophore availability
Source: ISME J. 2024 Jul 11;18(1):wrae123. doi: 10.1093/ismejo/wrae123 (PMC11296517; doi:10.1093/ismejo/wrae123)
Supplement: Siderophores_Supplementary_Figures_Corrected [file siderophores_supplementary_figures_corrected.pdf]

Table S1. List of human nasal isolates and their growth conditions.

Species	Name	Source	Over-night cultures			Spott-Assay
			Cultivation	Media	Inc Time	Inc Time
Bacillus cereus	44VPs_B5	(22)	aerobic	TSB	24 h	1 d
	45MNs_B5	(22)	aerobic	TSB	24 h	1 d
	89VPs_B11	(22)	aerobic	TSB	24 h	1 d
Bacillus mycoides	50MNt_Sm12	(22)	aerobic	TSB	24 h	1 d
Citrobacter koseri	10VAs_B1	(22)	aerobic	TSB	24 h	1 d
	44VAs_B2	(22)	aerobic	TSB	24 h	1 d
	9VAs_B2	(22)	aerobic	TSB	24 h	1 d
Corynebacterium accolens	10VAs_B6	(22)	aerobic	BHI-Tween	48 h	2 d
	50VPs_B7	(22)	aerobic	BHI-Tween	48 h	2 d
	63VAs_B8	(22)	aerobic	BHI-Tween	48 h	2 d
	83VAs_B5	(22)	aerobic	BHI-Tween	48 h	2 d
Corynebacterium aurimucosum	12UNs_B6	(22)	aerobic	BHI-Tween	48 h	2 d
	81VAs_KB1	(22)	aerobic	TSB	48 h	2 d
Corynebacterium hesseae	10VPs_Sm8	(22)	aerobic	TSB	24 h	2 d
Corynebacterium kroppenstedtii	82VAs_B6	(22)	aerobic	BHI-Tween	48 h	3 d
Corynebacterium propinquum	63VAs_B4	(22)	aerobic	BHI-Tween	48 h	2 d
	80VAs_KB2b	(22)	aerobic	BHI-Tween	48 h	2 d
	83VAs_B4	(22)	aerobic	BHI-Tween	48 h	2 d
	8VAs_B3	(22)	aerobic	BHI-Tween	48 h	2 d
Corynebacterium pseudodiphtheriticum	*M10-37*	(23)	aerobic	BHI-Tween	48 h	2 d
	M8-43	(23)	aerobic	BHI-Tween	48 h	2 d
	P1.29	(23)	aerobic	BHI-Tween	48 h	2 d
	P2.34	(23)	aerobic	BHI-Tween	48 h	2 d
	P6. 3	(23)	aerobic	BHI-Tween	48 h	2 d
	44VPs_Sm3	(22)	aerobic	BHI-Tween	48 h	2 d
	87VAs_B4	(22)	aerobic	BHI-Tween	48 h	2 d
	90VAs_B3	(22)	aerobic	BHI-Tween	48 h	2 d
Corynebacterium simulans	50VAs_B5	(22)	aerobic	TSB	24 h	1 d
	50MNs_SDm2	(22)	aerobic	TSB	24 h	1 d
	81MNs_B1	(22)	aerobic	BHI-Tween	48 h	2 d
	88UNs_Sm6	(22)	aerobic	BHI-Tween	48 h	2 d
Corynebacterium tuberculostearicum	12VAs_B4	(22)	aerobic	BHI-Tween	48 h	2 d
	87VAs_B5	(22)	aerobic	BHI-Tween	48 h	2 d
	89VPs_B8	(22)	aerobic	BHI-Tween	48 h	2 d
Cutibacterium acnes	50VAs_Sa1	(22)	anaerobic	TSB	24 h	1 d
	83VAs_Sa3	(22)	anaerobic	TSB	24 h	1 d
	87VAs_SaT9	(22)	anaerobic	TSB	24 h	1 d
	89VAs_Sa2	(22)	anaerobic	TSB	24 h	1 d
Cutibacterium avidum	10VAs_Sa2	(22)	anaerobic	TSB	24 h	1 d
	63VAs_Sa1	(22)	anaerobic	TSB	24 h	1 d
	83VAs_Sa1	(22)	anaerobic	TSB	24 h	1 d
	89VAs_KBa2	(22)	anaerobic	TSB	24 h	1 d
Cutibacterium granulosum	50MNt_Sa3	(22)	anaerobic	TSB	24 h	1 d
	83VPs_KBa2	(22)	anaerobic	TSB	24 h	1 d
Dolosigranulum pigrum	9VAs_B4	(22)	aerobic	TSB	7 d	7 d
	90VAs_B6	(22)	aerobic	TSB	7 d	7 d
Finegoldia magna	*87VAs_Sa4*	(22)	anaerobic	TSB	4 d	4 d
	63VAs_Sa4	(22)	anaerobic	TSB	24 h	1 d
	83VAs_Sa6	(22)	anaerobic	TSB	48 h	1 d
Moraxella catarrhalis	44VAs_Sm4	(22)	aerobic	TSB	24 h	1 d
	80VAs_B4	(22)	aerobic	TSB	24 h	1 d
	90VAs_B10	(22)	aerobic	TSB	24 h	1 d
Peptoniphilus harei	82VAs_KBa3	(22)	anaerobic	TSB	48 h	3 d
Staphylococcus aureus	M11-28	(23)	aerobic	TSB	24 h	1 d
	M13-14	(23)	aerobic	TSB	24 h	1 d
	M15-5	(23)	aerobic	TSB	24 h	1 d
Staphylococcus capitis	*M9-48*	(23)	aerobic	TSB	24 h	1 d
	M11-5	(23)	aerobic	TSB	24 h	1 d
	M12-47	(23)	aerobic	TSB	24 h	1 d
	10VAs_KB2	(22)	aerobic	TSB	24 h	1 d
	44UNs_B2	(22)	aerobic	TSB	24 h	1 d
	50VAs_KB6	(22)	aerobic	TSB	24 h	1 d
Staphylococcus epidermidis	*M11-13*	(23)	aerobic	TSB	24 h	1 d
	M12-38	(23)	aerobic	TSB	24 h	1 d
	M14-1	(23)	aerobic	TSB	24 h	1 d
Staphylococcus hominis	50MNs_Sa6	(22)	aerobic	TSB	24 h	1 d
	89VPs_B7	(22)	aerobic	TSB	24 h	1 d
	9VPs_KB1	(22)	aerobic	TSB	24 h	1 d
Staphylococcus lugdunensis	*SL2*	(73)	aerobic	TSB	24 h	1 d
	SL9	(73)	aerobic	TSB	24 h	1 d
	SL13	(73)	aerobic	TSB	24 h	1 d
	SL27	(73)	aerobic	TSB	24 h	1 d
	SL37	(73)	aerobic	TSB	24 h	1 d
	SL57	(73)	aerobic	TSB	24 h	1 d
	SL62	(73)	aerobic	TSB	24 h	1 d
	SL71	(73)	aerobic	TSB	24 h	1 d
	SL72	(73)	aerobic	TSB	24 h	1 d
	SL81	(73)	aerobic	TSB	24 h	1 d
Mammaliicoccus sciuri	9VPs_Sm2	(22)	aerobic	TSB	24 h	1 d
Staphylococcus warneri	*M8-7*	(23)	aerobic	TSB	24 h	1 d
	M11-4	(23)	aerobic	TSB	24 h	1 d
	M15-28	(23)	aerobic	TSB	24 h	1 d
Streptococcus constellatus	*45UNs_KB5*	(22)	aerobic	TSB	7 d	7 d
Streptococcus dysgalactiae	81VAs_B8a	(22)	aerobic	TSB	48 h	3 d
	81VAs_KBa3a	(22)	aerobic	TSB	48 h	3 d
Streptococcus intermedius	84MNs_KBa6	(22)	aerobic	TSB	48 h	4 d
Streptococcus mitis	78MNs_B4	(22)	aerobic	TSB	3 d	4 d
	81UNs_Sa2a	(22)	aerobic	TSB	48 h	4 d
Streptococcus mutans	44UNt_B13	(22)	aerobic	TSB	48 h	3 d
Streptococcus oralis	44UNt_B6	(22)	aerobic	TSB	48 h	3 d
Streptococcus pneumoniae	80UNt_KB6	(22)	aerobic	TSB	48 h	3 d

Table S2. Oligonucleotides used in this study.

Designation	*5' – 3' sequence*	Purpose
Sfa_A	GATC**GGTACC**AGTATCTTTAGTTGATGATTCT	Deletion of *sfaDABC* in *S. aureus.*
Sfa_B	TAATATATTTATCAATAAGTCTAAGTTGACA	Deletion of *sfaDABC* in *S. aureus.*
Sfa_C	ACTTATTGATAAATATATTATAAGGTTATAGAATTTTATTAATCGT	Deletion of *sfaDABC* in *S. aureus.*
Sfa_D	CG**GAATTC**TTCTATTGGTAGTGTAAGTTGGATCA	Deletion of *sfaDABC* in *S. aureus.*
Sfa_Sc.F	TACTGTACCATTTTCATCTTTTACCGA	Screening of the *sfaDABC* mutation.
Sfa_Sc.R	TTTCATTAACATATCGCGAAATATCAAT	Screening of the *sfaDABC* mutation.
Sbn_A	CACCTAAAGAT**CCCGGG**ACGTCAGTGGC	Deletion of *sbnABCDEFGHI* in *S. aureus.*
Sbn_B	CATAGGTGTTTGCCCTACAGAATCTAAC	Deletion of *sbnABCDEFGHI* in *S. aureus.*
Sbn_C	CTGTAGGGCAAACACCTATGTAGTTTTACTGTGATGTTGAGGGAAATA	Deletion of *sbnABCDEFGHI* in *S. aureus.*
Sbn_D	AAATCAGCAA**GGTACC**ACCAATCAGCC	Deletion of *sbnABCDEFGHI* in *S. aureus.*
Sbn_Sc.F	CCTACAATCTTAGTATCTTTTAAATC	Screening of the *sbnABCDEFGHI* mutation.
Sbn_Sc.R	TTTAGATTCAAATTGATTCTCTGTAC	Screening of the *sbnABCDEFGHI* mutation.
pIMAY_F	TACATGTCAAGAATAAACTGCCAAAGC	Sequencing of pIMAY inserts.
pIMAY_R	AATACCTGTGACGGAAGATCACTTCG	Sequencing of pIMAY inserts.
11615_A	**GCGGATCCAGCTGCAG**CACCAATGAGCGACAC	Deletion of *R3064_11615* in *C. hesseae.*
11615_B	CATAAAAGGTAGCCTAACC	Deletion of *R3064_11615* in *C. hesseae.*
11615_C	GGCTACCTTTTATGTAACACGGGGGTAGCACC	Deletion of *R3064_11615* in *C. hesseae.*
11615_D	**AAGCTTCGAATTCTGCA**ATATCCCACGGGTTGAGCTG	Deletion of *R3064_11615* in *C. hesseae.*
11615_Sc.F	CGCCTAGCGCAATGTTGAAT	Screening of the *R3064_11615 mutation.*
11615_Sc.R	GTAATCGAGCAGGCGGTAGT	Screening of the *R3064_11615 mutation.*
03755_A	**GCGGATCCAGCTGCAGAATTCG**CGGACATCCTGGGCATCAACCAGG	Deletion of *R3064_03755* in *C. hesseae.*
03755_B	CATGGTCGTCTACTTTCGTGTTCGAGTTTG	Deletion of *R3064_03755* in *C. hesseae.*
03755_C	CTCGAACACGAAAGTAGACGACCATGTAATGAACGCTCGACTTTCCGCGCA	Deletion of *R3064_03755* in *C. hesseae.*
03755_D	**AAGCTTCGAATTCTGCAGCTGGACGATG**GTCTTGCCCTGTTCCT	Deletion of *R3064_03755* in *C. hesseae.*
03755_Sc.F	AATTCCCCCTTAACCCCGC	Screening of the *R3064_03755 mutation.*
03755_Sc.R	AGGGTAGTTTCACAGCACGA	Screening of the *R3064_03755 mutation.*
pJSC_F	TGCAGAATTCGAAGCTT	Amplification of pJSC232 backbone for SLIC cloning.
pJSC_R	CTGCAGCTGGATCCGC	Amplification of pJSC232 backbone for SLIC cloning
pJS_C Sc.F	CAAGGACAATTAACAGTTAACAAATAA	Sequencing of pJSC232 inserts.
pJSC_Sc.R	CGTTTCCCGTTGAATATGGCTCAT	Sequencing of pJSC232 inserts.

* Restrictions sites and sequences overlapping with the plasmid backbone to allow SLIC are indicated in bold.

Table S3. SirA and HtsA homologues in selected nasal commensals.

Species and strain designation	*S. aureus* receptor	Commensal Ortholog Protein ID	Identity (%)
Corynebacterium hesseae - 10VPs_Sm8	HtsA	R3O64_11615	26
		R3O64_03755	26
	SirA	R3O64_11615	34
		R3O64_03755	27
Citrobacter koseri - 44VAs_B2	HtsA	R3O63_20740	39
		R3O63_18180 (*fhuD*)	28
	SirA	R3O63_20740	23
Bacillus cereus - 45MNs_B5	HtsA	R3O67_00955	31
		R3O67_08985	33
		R3O67_21210	29
	SirA	R3O67_00955	40
		R3O67_21210	37
Corynebacterium simulans - 50MNs_SDm2	HtsA	R3O68_02125	27
		R3O68_05280 (*fepB*)	24
	SirA	R3O68_05280 (*fepB*)	28
Mammaliicoccus sciuri - 9VPs_Sm2	HtsA	R3O66_01910	46
	SirA	R3O66_01220	40
Corynebacterium pseudodiphtheriticum - 90VAs_B3	HtsA	R3O65_07865	21
	SirA	R3O65_07865	30
		R3O65_08540	62

Table S4. Antismash-based prediction of biosynthetic gene clusters in selected nasal commensal isolates.

Species	Cluster	Function	Known Similarity	Identity %	Siderophore class
Corynebacterium hesseae 10VPs_Sm8	5.1	T1PKS		--	
	6.1	NRPS-like	Dechlorocuracomycin	8	
			Desotamide	9	
	11.1	terpene	Carotenoid	25	
Citrobacter koseri 44VAs_B2	1.1	siderophore	Aerobactin	77	Hydroxamate
	2.1	NRPS	Turnerbactin	30	Catecholate
	5.1	thiopeptide	O-antigen	14	
	6.1	NRPS, T1PKS	Yersiniabactin	16/100	Phenolate
	7.1	arylpolyene	APE Ec	94	
Bacillus cereus 45MNs_B5	2.1	NRPS		--	
	2.2	NRPS	Bacillibactin	100	Catecholate
	2.3	siderophore	Petrobactin	100	Catecholate
	3.1	bacteriocin			
		LAP			
	5.1	bacteriocin			
	6.1	terpene	Molybdenum cofactor	17	
	8.1	bacteriocin			
	12.1	NRPS-like			
	14.1	betalactone	Fengycin	40	
	14.2	bacteriocin			
Corynebacterium simulans 50MNs_SDm2	3.1	T1PKS		--	
	3.2	NRPS-like	Desotamide	9	
	3.3	NRPS	Glycopeptidolipid	12	
	4.1	bacteriocin			
	6.1	terpene	Carotenoid		
Mammaliicoccus sciuri 9VPs_Sm2	2.1	bacteriocin			
	3.1	terpene			
	3.2	siderophore			unknown
	7.1	lathipeptide	Nukacin ISK-1	18	
Corynebacterium pseudodiphtheriticum 90VAs_B3	5.1	terpene			
	5.2	T1PKS		--	
	8.1	NRPS	Cahuitamycins	8	

Corynebacterium hesseae 10VPs_Sm8	5.1	T1PKS		--	
	6.1	NRPS-like	Dechlorocuracomycin	8	
			Desotamide	9	
	11.1	terpene	Carotenoid	25	
Citrobacter koseri 44VAs_B2	1.1	siderophore	Aerobactin	77	Hydroxamate
	2.1	NRPS	Turnerbactin	30	Catecholate
	5.1	thiopeptide	O-antigen	14	
	6.1	NRPS, T1PKS	Yersiniabactin	16/100	Phenolate
	7.1	arylpolyene	APE Ec	94	
Bacillus cereus 45MNs_B5	2.1	NRPS		--	
	2.2	NRPS	Bacillibactin	100	Catecholate
	2.3	siderophore	Petrobactin	100	Catecholate
	3.1	bacteriocin			
		LAP			
	5.1	bacteriocin			
	6.1	terpene	Molybdenum cofactor	17	
	8.1	bacteriocin			
	12.1	NRPS-like			
	14.1	betalactone	Fengycin	40	
	14.2	bacteriocin			
Corynebacterium simulans 50MNs_SDm2	3.1	T1PKS		--	
	3.2	NRPS-like	Desotamide	9	
	3.3	NRPS	Glycopeptidolipid	12	
	4.1	bacteriocin			
	6.1	terpene	Carotenoid		
Mammaliicoccus sciuri 9VPs_Sm2	2.1	bacteriocin			
	3.1	terpene			
	3.2	siderophore			unknown
	7.1	lathipeptide	Nukacin ISK-1	18	
Corynebacterium pseudodiphtheriticum 90VAs_B3	5.1	terpene			
	5.2	T1PKS		--	
	8.1	NRPS	Cahuitamycins	8	

Table S5. Bacterial strains isolated from the nose of cotton rats.

Species *	Strain designation	Siderophore-production**
Aerococcus viridans	CR_01	No
Staphylococcus xylosus	CR_02	Yes
Escherichia coli	CR_03	No
Enterobacter cloacae	CR_04	Yes
Haemophilus influenzae	CR_05	No
Rothia nasimurium	CR_06	No
Corynebacterium casei	CR_07	No
Micrococcus luteus	CR_08	No
Bacillus megaterium	CR_09	Yes
Enterococcus faecalis	CR_10	No
Staphylococcus sciuri	CR_11	No
Corynebacterium aurimucosum	CR_12	No

* Identified by MALDI-TOF-MS ** Positive in the CAS-assay

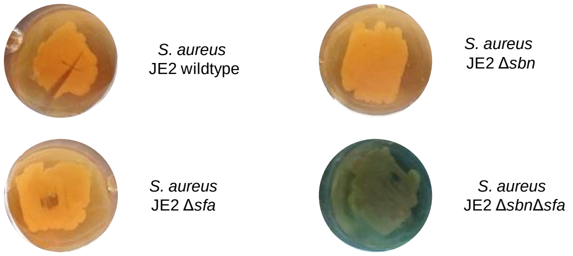

Fig. S1: Siderophore production by *S. aureus* USA300 JE2 and isogenic mutants.
Bacterial isolates were spotted on BHI-EDDHA agar in 24 well plates and incubated for one week at 37°C**.** After incubation wells were overlaid with CAS-containing top agar. Color change to yellow indicates siderophore production and was assessed 4 hours after the overlay.

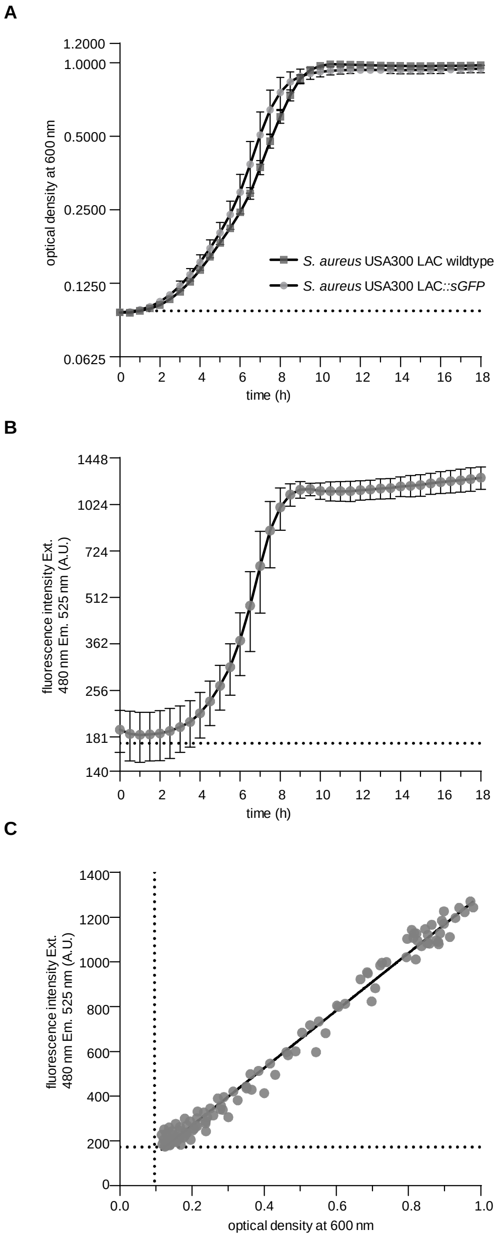

Fig.S2: Growth of *S. aureus* USA300 LAC*::sGFP*.
Growth curves in iron-limited medium: *S. aureus* USA 300 LAC*::sGFP and S. aureus* USA300 LAC WT were inoculated to an optical density OD 0.01 and grown for 18 h in 500 µl iron-limited medium (1x RPMI, 1% casamino acid, 10 µM EDDHA, 100 µg holo-transferrin) at 37°C under constant shaking in the Tecan Spark ® 10M multimode microplate reader. Growth was monitored via optical density at 600 nm and fluorescence intensity of the GFP signal (Ext. 480 nm and Em. 525 nm). The dotted line indicates the media control. Mean and SD of three to eight experiments is shown. **A)** Shows a comparison of the OD_{600} growth curves obtained by the *S. aureus* USA300 LAC wildtype and *S. aureus* USA300 LAC*::sGFP,* and **B)** shows the *S. aureus* USA 300 LAC*::sGFP* curve generated through the fluorescent signal. ***C)*** Correlation of OD_{600} and GFP signal (2,5 h – 11 h of the growth) indicate a linear relationship (formula y=12.54+1284*x; R^2 = 0.98).

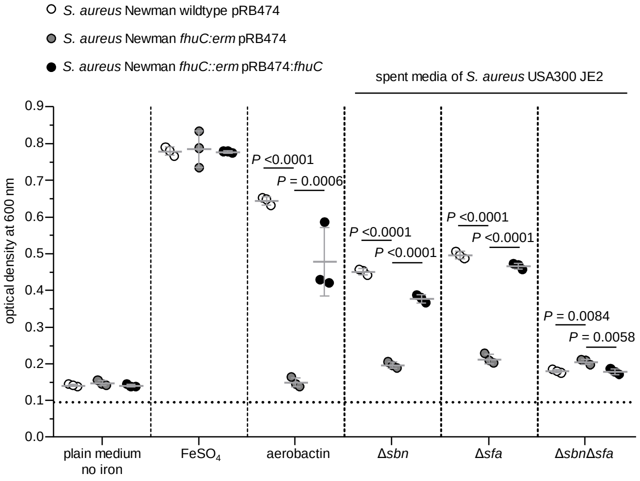

Fig. S3. Iron dependent growth of *S. aureus* Newman[strepR] ***fhuC::erm***
Strains were grown in the presence of 20 µM $FeSO_4$, 200 nM aerobactin, 9.1% spent medium of *S. aureus* USA300 JE2 Δ*sfa* (source of SF-B), 5,7% spent medium of *S. aureus* USA300 JE2 Δ*sbn* (source of SF-A) as a sole source of iron. 500 µl of cultures were inoculated to an OD_{600}= 0.05 in 48 well plates and OD_{600} was measured after 20 h using an Epoch1 plate reader. Mean and SD of three experiments are shown. Statistical analysis was performed using one-way ANOVA (*P* <0,0001) with subsequent multiple comparison.
